# The Use of Nanotrap Particles Technology in Capturing HIV-1 Virions and Viral Proteins from Infected Cells

**DOI:** 10.1371/journal.pone.0096778

**Published:** 2014-05-12

**Authors:** Elizabeth Jaworski, Mohammed Saifuddin, Gavin Sampey, Nazly Shafagati, Rachel Van Duyne, Sergey Iordanskiy, Kylene Kehn-Hall, Lance Liotta, Emanuel Petricoin, Mary Young, Benjamin Lepene, Fatah Kashanchi

**Affiliations:** 1 National Center for Biodefense & Infectious Diseases, George Mason University, Manassas, Virginia, United States of America; 2 Department of Microbiology, Immunology, and Tropical Medicine, George Washington University, Washington, District of Columbia, United States of America; 3 Center for Applied Proteomics and Molecular Medicine, George Mason University, Manassas, Virginia, United States of America; 4 Division of Infectious Diseases, Georgetown University, Washington, District of Columbia, United States of America; 5 Ceres Nanosciences, Manassas, Virginia, United States of America; National Institutes of Health, United States of America

## Abstract

HIV-1 infection results in a chronic but incurable illness since long-term HAART can keep the virus to an undetectable level. However, discontinuation of therapy rapidly increases viral burden. Moreover, patients under HAART frequently develop various metabolic disorders and HIV-associated neuronal disease. Today, the main challenge of HIV-1 research is the elimination of the residual virus in infected individuals. The current HIV-1 diagnostics are largely comprised of serological and nucleic acid based technologies. Our goal is to integrate the nanotrap technology into a standard research tool that will allow sensitive detection of HIV-1 infection. This study demonstrates that majority of HIV-1 virions in culture supernatants and Tat/Nef proteins spiked in culture medium can be captured by nanotrap particles. To determine the binding affinities of different baits, we incubated target molecules with nanotrap particles at room temperature. After short sequestration, materials were either eluted or remained attached to nanotrap particles prior to analysis. The unique affinity baits of nanotrap particles preferentially bound HIV-1 materials while excluded albumin. A high level capture of Tat or Tat peptide by NT082 and NT084 particles was measured by western blot (WB). Intracellular Nef protein was captured by NT080, while membrane-associated Nef was captured by NT086 and also detected by WB. Selective capture of HIV-1 particles by NT073 and NT086 was measured by reverse transcriptase assay, while capture of infectious HIV-1 by these nanoparticles was demonstrated by functional transactivation in TZM-bl cells. We also demonstrated specific capture of HIV-1 particles and exosomes-containing TAR-RNA in patients' serum by NT086 and NT082 particles, respectively, using specific qRT-PCR. Collectively, our data indicate that certain types of nanotrap particles selectively capture specific HIV-1 molecules, and we propose to use this technology as a platform to enhance HIV-1 detection by concentrating viral proteins and infectious virions from infected samples.

## Introduction

Human immunodeficiency virus type 1 (HIV-1) virions possess 9.7 kb dimeric positive-sense single-stranded RNA genome, which contains a total of nine genes, structural (env, gag, pol), regulatory (tat, rev) and accessory (nef, vif, vpr, vpu) [1]. HIV-1 transmission occurs via contact with infected bodily fluids during sexual intercourse, childbirth, breast-feeding, blood transfusion or intravenous drug use [2], [3], [4], [5], [6]. Since its discovery in 1981, HIV-1 caused more deaths than any other single infectious disease [7], [8]. As of 2011, an estimated 25 million people have died of acquired immunodeficiency syndrome (AIDS) caused by HIV-1 infection, and 34 million are currently living with HIV-1 infection worldwide; and there is not yet an effective vaccine [7]. Moreover, 2.5 million new infections worldwide and approximately 50,000 in the United States alone have been reported each year [7], [8], [9]. Also, a substantial number of newly infected individuals are not aware of their HIV-1 positive status [8], [10], [11]. Although long-term highly active antiretroviral therapy (HAART) can halt the virus replication in blood to an undetectable level, discontinuation of therapy rapidly increases virus burden [12], [13]. Moreover, patients under HAART frequently develop various metabolic disorders and also HIV-associated neuronal disease [14], [15], [16], [17], [18]. Today, the main challenge of HIV-1 research is the elimination of the residual virus in infected individuals [12], [13], [19].

Tat is an essential regulatory protein that directs efficient elongation of the HIV-1 genome. It binds to an RNA stem-loop structure, the trans-activating response element (TAR) at the 5′ ends of HIV-1 transcripts, and recruits a positive transcription elongation complex (P-TEFb) to increase the production of full-length viral RNA [20], [21]. The minimum TAR binding sequence of Tat has been mapped to a basic domain of 10 amino acids, comprising mostly Arg and Lys residues, and the regulatory activity requires the 47 N-terminal residues, which interact with components of the transcription complex and function as a transcriptional activation domain [22]. In addition to HIV-1 transcription, active extracellular secretion of Tat has been reported to influence various cellular genes expression [23], [24]. The accessory proteins especially Nef may be dispensable for virus replication *in vitro* but essential for HIV-1 pathogenesis *in vivo* [25]. Nef is a myristoylated protein, localizes to endosomal and plasma membranes and affects many cellular pathways including cellular activation, cell survival and apoptosis [26]. Nef has also been shown to play an active role in modulating immune activation and long-term maintenance of viral replication *in vivo* [27].

Current HIV-1 diagnostics are largely based on the presence of anti-viral antibody in the blood [28], [29], [30]. Of particular concern is that elevated viral levels during acute infection contribute to as much as 26 times more efficient sexual transmission than that of chronic infection [3], [31]. However, due to lack of HIV-specific antibodies in early acute infection, many of the currently employed serological diagnostics are not applicable for detection of the status of HIV-1 infection [32], [33], [34]. Alternative methods for detection of early HIV-1 infection rely on the measurement of viral RNA in blood samples by polymerase chain reaction (PCR) [35]. While HIV-1 nucleic acid amplification assays are extremely sensitive and can reliably detect HIV-1 very early in infection, the fact that the false-positive rates could be as high as 1%, and their use for routine clinical HIV-1 screening is impeded by their high cost [33]. Though much of these methods are automated and designed for high throughput, several other disadvantages include requirement of trained personnel and complex equipment, and the propensity for contamination [35], [36]. Apart from the above limitations in regard to early diagnosis of HIV-1 infection, a number of highly sensitive serological and nucleic acid based technologies have been developed for both *in vivo* diagnosis and laboratory research of HIV-1. Nevertheless, to our knowledge, no means currently exist to capture, sequester and concentrate HIV-1 virions or proteins.

Our innovative approach employs multifunctional hydrogel nanotrap particles to capture and enrich a variety of target molecules from both *in vivo* and *in vitro* samples [37]. For example, several groups have recently utilized these nanotrap particles to capture various biomarkers including bacterial antigen of Lyme disease and human growth hormone from urine [38], [39]; Bak in the detection of melanoma as well as components of the peptidome associated with ovarian and prostate cancer [40], [41]. Structurally, these particles are composed of high affinity aromatic baits containing core surrounded by a sieving shell [38]. Synthesis of these particles requires polymerization reactions using the reactants N-isopropyl acrylamide (NIPAm), N,N-Methylenebis-acrylamide (Bis) and either allylamine (AA), acrylic acid (AAc) or methacrylate (MA) (see Table 1). In some instances, vinyl sulfonic acid (VSA) monomers can be incorporated into the shell to improve the ability to exclude high abundance and high molecular weight analytes. Once the sieving shell is formed, specific organic baits are covalently attached to the core matrix [38]. The pores of the shell function to selectively permit the passage of smaller molecules below a predetermined size and actively exclude high abundance large proteins such as albumin. Lastly, the high affinity charge-based bait located in the inner core, dissociates and binds target molecules through electrostatic and hydrophobic interactions; sequestering them while concurrently preventing degradation [42].

**Table 1:** Structural characteristics of nanotrap particles*.

ID	Core Bait	Matrix	Shell	VSA in Shell	Target
NT073	Cibacron Blue F3GA	NIPAm-Bis-AA	Y	Y	HIV-1 virion, gp41
NT077	Acrylic Acid	NIPAm-Bis-AAc	Y	N	No binding to Tat
NT080	Reactive Red 120	NIPAm-Bis-AA	Y	N	Exosomal TAR-RNA, intracellular Nef, p24
NT081	Cibacron Brilliant Yellow	NIPAm-Bis-AA	Y	N	Minimum binding to Tat
NT082	Cibacron Blue F3GA	NIPAm-Bis-AA	Y	N	Exosomal TAR-RNA, Tat
NT083	Allylamine	NIPAm-Bis-AA	Y	N	Minimum binding to Tat
NT084	Acid Black 48	NIPAm-Bis-AAc	N	N	Tat, p24
NT085	Pigment Red	NIPAm-Bis-AAc	Y	Y	No binding to Tat
NT086	Methacrylate	NIPAm-Bis-MA	Y	Y	HIV-1 virion, membrane-associated Nef, gp41, minimum binding to Tat

*Nanotrap particles are homogenous hydrogel particles of about 700–800 nm in diameter that have a shell made of polymers of N-isopropylacrylamide (NIPAm) and co-monomers such as acrylic acid (AAc), allylamine (AA) and methacrylate (MA) with cross links of N,N'-methylenebisacrylamide (Bis) [37], [54]. Some of these shelled nanotrap particles are also coated with vinyl sulfonic acid (VSA) [38].

Here, we discuss a novel method by which HIV-1 antigens are captured and concentrated by nanotrap particles and then quantitated by standard assays such as western blot, RT assay, PCR or mass spectrometry. Additional advantages of this technology include flexibility to multiple buffering conditions and a short incubation at room temperature. We also demonstrate the specificity of several nanotrap particles in capturing of HIV-1 virions and individual proteins, including Tat and Nef.

## Materials and Methods

### Ethics Statement

All research involving human participants have been approved by The Georgetown University (GTU) Institutional Review Board (IRB) for The Women's Interagency HIV Study (WIHS) which covers viral and immunologic studies related to HIV-1 infection and its co-morbidities. All the participants provided written informed consent by signing consent forms to participate in viral load testing studies under WIHS, and they are on file. All participants were assigned a unique study number that is used as identification on all data sheets, stored specimens and laboratory requests. The participants' names and contact information are listed on a separate log that is kept with locator information in a locked cabinet in a locked room separate from the data file room. No reports or publications from the WIHS will include any individuals' identifying information. This consent procedure was approved by the GTU IRB for the WIHS study.

All animal works have been conducted according to the US national guidelines and approved by the George Mason University Institutional Animal Care and Use Committee.

### Cell lines

The uninfected Jurkat and HIV-1 infected J1.1 T-cells obtained from the NIH AIDS Research and Reference Reagent Program, were maintained in RPMI-1640 media containing 10% heat-inactivated fetal bovine serum (FBS), 1% L-glutamine, and 1% streptomycin/penicillin (Quality Biological, Gaithersburg, MD). A HeLa cell derivative TZM-bl cells, expressing CD4, CXCR4 and CCR5 surface receptors, and with a stably integrated firefly luciferase reporter gene under the control of an HIV-1 LTR [43], [44], was maintained in DMEM supplemented with 10% FBS, 1% L-glutamine, and 1% streptomycin/penicillin. All cells were incubated at 37°C in the presence of 5% CO_2_.

### Purified protein samples and antibodies

Full-length HIV-1 Tat protein (aa1-86) was prepared as described [45]. Briefly, Tat protein was expressed in E. coli. Bacterial cells were sonicated and clarified by centrifugation before chromatographed on a Sephacryl S-200 column, and then finally, purified on a reverse phase HPLC column. Transcriptionally inactive Tat peptide containing basic domain (aa36–72) [22] obtained from Peptide Technologies Corporation (Cat# 2369, Gaithersburg, MD), was tested for initial capture by nine different nanotrap particles (see Table 1). The molecular weight of this peptide is 3506.90 Da, and the amino acid sequence comprises of VCFTTAACSIAAGRKKRRORRRPPOGSQTHQVSLSKO. The purified T-tropic HIV-1 gp120 protein, *HXB120* was generously provided by Dr. Wen Yuan (University of Virginia). The monoclonal antibody to HIV-1 p24 (AG3.0) and gp41 (No. 98-6, and 50–69) were obtained through the NIH AIDS Research and Reference Reagent Program, contributed by Drs. Jonathan Allan and Susan Zolla-Pazner, respectively. The monoclonal anti-Nef antibody was obtained through the NIH AIDS Reagent Program, contributed by Dr. James Hoxie.

### Preparation of whole cell extract

Actively growing cells were pelleted, washed twice with PBS (without Ca^2+^ and Mg^2+^) and resuspended in lysis buffer (50 mM Tris-HCl, pH 7.5; 120 mM NaCl; 5 mM EDTA; 0.5% NP-40; 50 mM NaF; 0.2 mM Na_3_VO_4_; 1 mM DTT; and one protease cocktail tablet/50 ml) before incubating on ice for 20 min with gentle vortexing. The whole cell extracts (WCE) were centrifuged at 10,000 rpm for 10 min at 4°C, and protein concentrations were determined using the Bradford protein assay (Bio-Rad, Hercules, CA).

### Synthesis of nanotrap particles

The nanotrap particles provided by Ceres Nanosciences (Manassas, VA) were synthesized as previously described [38], [42]. Briefly, the poly-N-isopropylacrylamide (pNIPAm) based core with incorporated acrylic acid (AAc) was synthesized via precipitation polymerization, with N'N-methylene bisacrylamide (BIS) serving as the crosslinker. The core particles were further functionalized by covalently immobilizing affinity dyes onto the polymer matrix. For nanotrap particles with a core-shell architecture, the crosslinked pNIPAm-based core is encapsulated within a crosslinked shell. The crosslinked shell can be inert and consist only of crosslinked pNIPAm or contain chemical moieties such as vinyl sulfonic acid (VSA).

### Nanotrap particles binding conditions

Each target analyte was captured by nanotrap particles as described below. Briefly, a 30% slurry (30 µl) of each type of nanotrap particles was washed twice with specific working buffer and then incubated with target molecule spiked in 100 µl of appropriate binding buffer for 30 min at room temperature. After 2–3 washes (each 5 min) nanotrap particles containing captured molecules were resuspended in specific buffer based on the downstream assays used as described below:

To capture purified proteins, aliquots of Tat_36–72_ peptide (0.1, 1, 10 µg), full-length Tat (0.1 µg) or gp120 (1 µg) was spiked into 100 µl of FBS-supplemented RPMI and incubated with the slurry of each nanotrap particles for 30 min at room temperature. Nanotraps containing each captured molecules were washed in autoclaved deionized water (200 µl), resuspended in Laemmli buffer, resolved on SDS-PAGE gel and analyzed by western blot using anti-Tat antibody or HIV-1 immune serum. In the absence of purified native Nef, p24 or gp41 protein, HIV-1 infected J1.1 WCE (30 µg) or supernatant was spiked into TNE_150_ buffer without detergent and similarly incubated with nanotrap particles before analyzing elutes by western blot using specific antibodies to HIV-1 nef, p24 or gp41. To determine whether simpler form of proteins interacts better with nanotrap particles, Tat protein was treated with 10 mM DTT for 30 min at 37°C before incubating with particles.

To capture whole HIV-1 particles, 30 µl slurry of nanotrap particles was incubated with 1 ml of J1.1 cell supernatants for 30 min at room temperature. Particles were washed by centrifugation (15,000 rpm) for 5 min to remove unbound virus and then incubated with reverse transcriptase (RT) assay reaction buffer containing 50 mM Tris–HCl, 1 mM DTT, 5 mM MgCl_2_, 20 mM KCl, 0.1% Triton, poly(A) (1 U/ml), poly(dT) (1 U/ml) and [^3^H]TTP. The mixture was incubated overnight at 37°C, and 10 µl of the reaction mix was spotted on a DEAE Filter mat paper (PerkinElmer, Shelton, CT, USA), washed four times with 5% Na_2_HPO_4_ and three times with water, and then dried completely. RT activity was measured in a Betaplate counter (Wallace, Gaithersburg, MD).

To determine the utility of nanotrap particles for capturing HIV-1 virions directly from patients' samples a slurry of nanotrap particles was incubated in a final volume of 1 ml PBS containing 100 µl serum samples from 4 HAART-treated subjects and 2 uninfected control donors. After incubation at room temperature for 30 min, the nanotrap particles were washed and the level of nanotrap particles-bound virus was quantified by qRT-PCR using primers specific for HIV-1 unspliced RNA as described [46].

To capture live HIV-1 by nanotrap particles, J1.1 supernatants (1, 10, 100 µl) diluted to 1 ml in complete media, were either untreated or added to 50 µl slurry of NT086 particles before incubating for one hour with gentle rotation at room temperature. Nanotrap particles were washed to remove unbound virus by centrifugation for 10 min and then resuspended in 100 µl of complete media before adding directly to a monolayer of TZM-bl cells (5×10^4^, 50% confluent) in microtiter plates. The J1.1 cell supernatants were also incubated with TZM-bl cells without prior treatment with nanotrap particles. After 48 hrs post incubation at 37°C, cells were lysed and luciferase activity was measured using BrightGlo Luciferase Assay (Promega) following manufacturer's instructions. Luminescence was measured with Promega GloMax Multi Detection System. Similarly, NT073 nanoparticles were also used to capture live virus using the above TZM-bl system. After 96 hr post culture, luciferase activity was measured by the BrightGlo Luciferase Assay as above.

### Staining and western blot

Materials were eluted from the nanotrap particles in Laemmli buffer (15 µl), frozen at −20°C for 10 min, heated at 95°C for 5 min, and spun (15,000 rpm) for 5 min. The entire volume was loaded onto a 4–20% Tris-Glycine gel, run at 200 V, and transferred onto nitrocellulose membranes. Gels were Coomassie stained with 40% methanol, 7% glacial acetic acid, and Coomassie Brilliant Blue (Bio-Rad, R-250). Membranes were blocked with D-PBS containing 0.1% Tween-20 and 3% BSA, and incubated overnight at 4°C with the appropriate primary antibody (α-Tat, α-Nef, α-p24, α-gp41, α-CD63, α-Alix, or α-Actin). Membranes were then incubated with the appropriate HRP-conjugated secondary antibody and developed next day.

### Detection of TAR-RNA in nanotrap particles-bound exosomes by RT-PCR

We have shown previously that exosomes prepared from HIV-1 infected J1.1 cell supernatant contain TAR-RNA [46]. To determine whether the specific nanotrap particles that efficiently capture high levels of J1.1 cell-derived HIV-1 could also capture exosomes that are derived from the J1.1 cell supernatants, exosomes preparations were similarly incubated with a slurry of 5 different nanotrap particles. After washing, total RNA was extracted via the addition of Trizol Reagent (Invitrogen) (750 µl) to the pelleted particles-bound exosome materials. After incubation for 5 min at room temperature, chloroform (200 µl) was added to the samples and further incubated for 3 min. Samples were centrifuged (12,000×g) for 15 min at 4°C. To the aqueous phase, 100% isopropanol (350 µl) was added and centrifuged (12,000×g) for 10 min at 4°C. The pellet was washed with 75% ethanol (750 µl) and centrifuged. The RNA pellet was air dried and reconstituted in 1X TE buffer, heated to 65°C for 10 min and stored at −20°C. Following RNA extraction, cDNA synthesis was completed using the iScript Select cDNA Synthesis Kit (Bio-Rad). The newly synthesized cDNA (10 µl) was amplified by qRT-PCR using TAR-RNA specific primers [46], and copy numbers of nanotrap particles-bound TAR-RNA compared.

### Quantitation of HIV-1 particles bound per microgram of nanoparticles

A 100 µl aliquot of the dual-tropic HIV-1 89.1 containing 10 or 1 ng p24/ml in RPMI-1640 medium was incubated with a pellet of 0.02, 0.2 or 2.0 mg of NT086 nanoparticles for 30 min at room temperature. The virus bound-nanoparticles were washed three times with RPMI-1640 by centrifugation, and subjected to RNA isolation by Trizol Reagent (Invitrogen, Carlsbad, CA). Purified RNA was then converted to cDNA by incubating with Oligo-dT primers using GoScript Reverse Transcription System (Promega, Madison, WI). PCR reaction mixtures were prepared using 2 µl of undiluted, 10^−1^ or 10^−2^ diluted cDNA, 300 nm of each primer: Gag1483-F (5′-AAGGGGAAGTGACATAGCAG-3′) and Gag1625-R: (5′-GCTGGTAGGGCTATACATTCTTAC-3′), and the iTaq Universal SYBR Green Supermix (Bio-Rad, Hercules, CA), to amplify 143 nt fragments of HIV-1 gag gene. Serial dilutions of DNA from 8E5 cells (a CEM-derived cell line containing a single copy of HIV-1 LAV provirus per cell) were used as the standards. Quantitative real-time PCR reactions were then carried out in triplicate using the CFX96 Real-Time PCR System (BioRad) and SFX Manager Software ver. 2.0.

## Results

### Nanotrap particles capture HIV-1 Tat peptide, full-length Tat, Nef, and p24

To determine the target selectivity for HIV-1 virions or viral proteins, 9 different hydrogel nanotrap particles (from Ceres Nanoscience) each containing specific bait such as Cibacron Blue, Allylamine or Methacrylate, were used in this study (Table 1). One nanotrap particle (NT084) did not contain the outer shell, and three of the particles (NT073, NT085 and NT086) contained VSA incorporated into the shell. The nanotrap particles have submicron dimensions and contain a variety of functional baits in the core. As shown in Table 1, each nanotrap particle showed specific selectivity for capturing either whole HIV-1 virion or viral protein.

**Figure 1:**
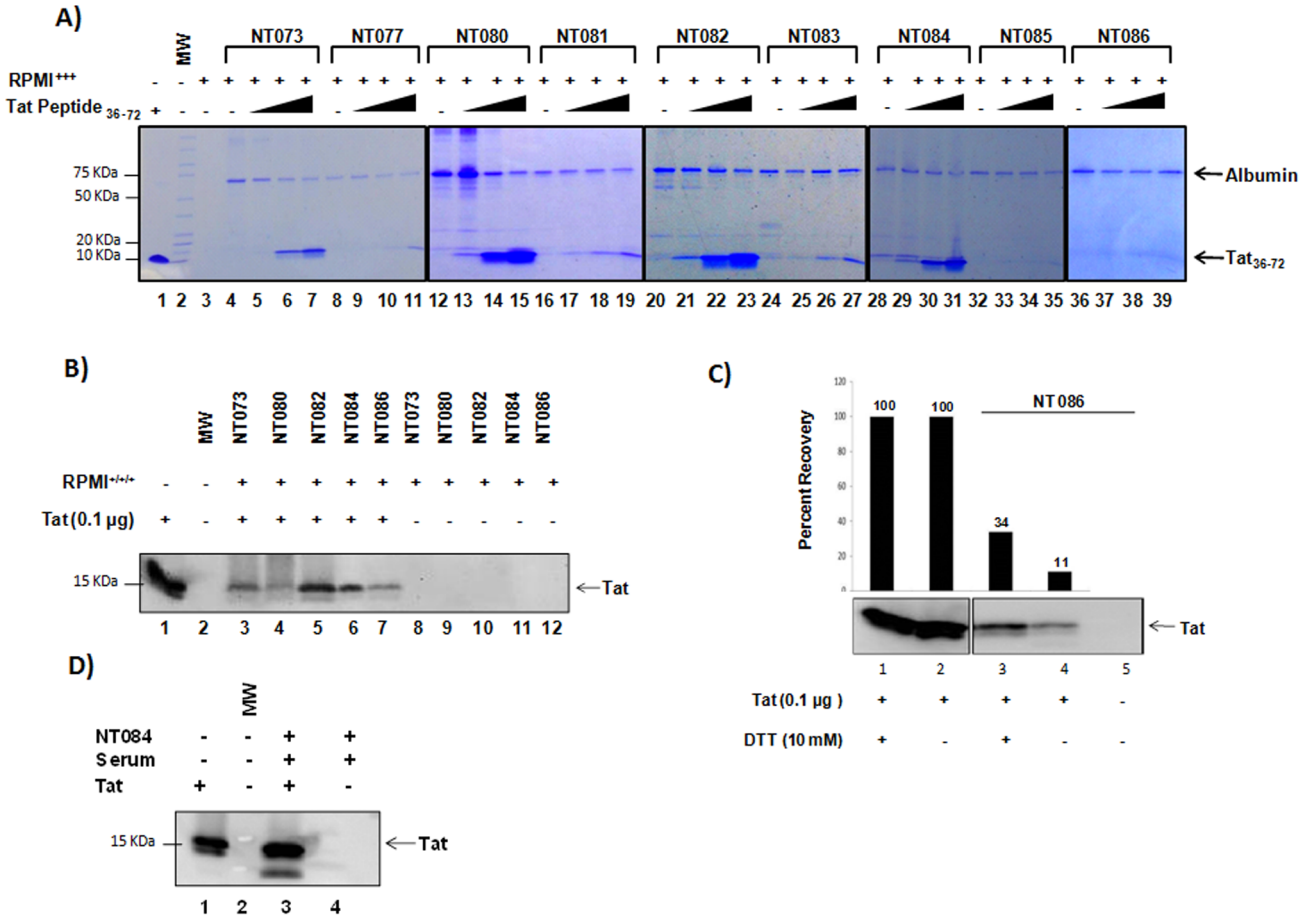
Capture of Tat protein by nanotrap particles. (**A**) Tat_36–72_ peptide was spiked into RPMI-1640 containing FBS, antibiotics and glutamine (RPMI+++) at 0.1 µg (lanes 5, 9, 13, 17, 21, 25, 29, 33, 37), 1 µg (lanes 6, 10, 14, 18, 22, 26, 30, 34, 38), and 10 µg (lanes 7, 11, 15, 19, 23, 27, 31, 35, 39). A 30% slurry of nanotrap particles prepared in unsupplemented RPMI was incubated with RPMI containing Tat_36–72_ for 30 min at room temperature. Eluted materials (15 µl) were then coomassie stained for the presence of captured Tat peptide. (**B**) Full-length Tat protein spiked in RPMI-1640 (+++) (100 µl) was captured by a 30% slurry of each nanotrap particle in unsupplemented RPMI (lanes 3–7), separated on SDS-PAGE and detected by Western blot using anti-tat antibody. Nanotrap particles incubated with RPMI+++ without Tat were used as background control (lanes 8–12). (**C**) Full-length Tat was pre-treated with DTT at 37°C for 30 min (lanes 1 and 3) and incubated with NT086 (lane 3). Percent recovery Tat capture under reducing conditions was determined from raw densitometry counts of the western blot. (**D**) Tat protein (1 µg) was spiked into uninfected patient serum (100 µl), incubated with NT084 (lane 3), and subsequent Tat capture determined by western blot.

In order to determine if nanotrap particles could bind HIV-1 proteins, we initially screened all 9 different nanotrap particles for their ability to captureTat_36–72_ peptide. This core domain of Tat is responsible for the binding of TAR-RNA, the RNA regulatory element within the HIV-1 LTR, and subsequent activation of transcription of the proviral genome. Structurally, this region is composed of two domains, a hydrophobic core and a glutamine-rich domain, and flanked by cysteine-rich domains [47]. First, we spiked increasing concentrations of Tat_36-72_ peptide (0.1–10 µg) into FBS-supplemented RPMI and then incubated with particle slurry. A coomassie stain of materials eluted from nanotrap particles revealed that NT073, NT080, NT082, and NT084 (Fig. 1A, lanes 5–7, 13–15, 21–23, 29–31) successfully captured Tat_36–72_ peptide. The nanotrap particles NT077, NT085, and NT086 failed to capture Tat_36–72_ peptide (lanes 9–11, 33–35, 37–39).

To confirm the ability of NT073, NT080, NT082, and NT084 to capture full-length Tat protein, slurry of each nanotrap particle was incubated with full-length Tat (Fig. 1B). Since NT086 was unable to capture Tat_36–72_, we anticipated this particle would fail to capture the full-length protein. Thus this nanotrap particle served as our negative control. Western blot analysis revealed that NT073, NT082 and NT084 successfully captured full length Tat (Fig. 1B lanes 3, 5 and 6), while NT080 and NT086 (lanes 4 and 6) recovered Tat, albeit at lower levels. Interestingly, these results present a discrepancy in the binding of NT086 to Tat_36–72_ and full-length Tat. We therefore pre-treated Tat with DTT to reduce and unfold the protein, and completed the nanotrap particles-mediated pulldown (Fig. 1C). We observed a better recovery in the binding of the reduced Tat (34%) as compared to the native protein (11%) by NT086 (Fig. 1C, compare lanes 3 and 4). However, the overall binding of Tat by this nanotrap particle was still low.

We then explored the ability of the nanotrap particles to capture HIV-1 Tat protein from a complex physiologically relevant media. We spiked full-length Tat protein into serum collected from an uninfected donor. Since our previous data indicated that NT084 efficiently captured Tat in cell culture media (Fig. 1A, 1B), we utilized this nanotrap particle for the pulldown of Tat from serum. Western blot analysis revealed that NT084 also recovered Tat efficiently from serum compared to the input (Fig. 1D, compare lanes 1 and 3). Collectively, our results demonstrate that nanotrap particles especially NT084 successfully captured HIV-1 Tat protein from cell culture media as well as complex fluids such as serum.

### Nanotrap particles capture HIV-1 Nef protein more efficiently than p24 capsid protein

**Figure 2:**
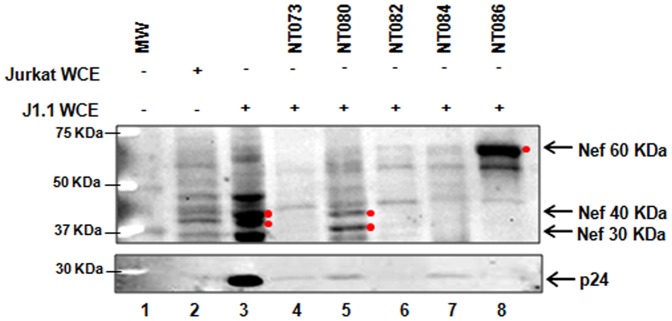
Capture of HIV-1 Nef and p24 capsid proteins by nanotrap particles. Aliquots of infected J1.1 whole cell extract (WCE) (100 µg) in 100 µl of water was incubated with 75 µl of a 30% slurry of nanotrap particles in TNE buffer without detergent for 30 min (lanes 4–8). Nef binding capacity of each nanotrap particle was subsequently determined by western blot using α-Nef antibody (upper panel). The binding of p24 to nanotrap particles was also determined by western blot using α-p24 antibody (lower panel). Both Jurkat and J1.1 WCE were used as background controls (lanes 2, 3) without enrichment by nanotrap particles.

After having explored the binding capabilities of the nanotrap particles to HIV-1 Tat, we next asked whether these particles could capture other HIV-1 proteins in their native forms. Since our previous results indicated that NT073, NT080, NT082, NT084, and NT086 captured Tat protein, we employed these nanotrap particles for subsequent experiments. Due to unavailability of functionally native Nef, p24 and gp41 proteins, we explored the capacity of these particles to capture HIV-1 proteins from the infected J1.1 cells. The WCE of J1.1 cells (containing native viral proteins prior to packaging) resuspended into TNE_150_ buffer without detergent was added to a standard slurry of nanotrap particles NT073, NT080, NT082, NT084 or NT086. Materials eluted from each nanotrap particle were analyzed for the presence of Nef and p24 proteins by western blotting. Our results demonstrate that NT080 captured the intracellular 30–40 kDa forms of Nef (Fig. 2A, lane 5), while NT086 captured the membrane-associated 60 kDa form of Nef (Fig. 2A, lane 8). We also observed that NT073, NT080, and NT084 bound very low levels of p24, while NT082 and NT086 completely failed to capture p24 (Fig. 2, lower panel). The higher binding affinity of nanotrap particles especially NT086 for membrane-derived Nef and NT080 for both intracellular Nef and p24 proteins further suggest a selectivity of nanotrap particles for specific target molecules.

Previously we were able to influence the binding capability of NT086 by reducing Tat protein (Fig. 1C). We therefore asked if the presence of a reducing agent would enhance the binding affinities of the nanotrap particles for Nef. We incubated J1.1 WCE in the presence of DTT for 30 min at 37°C and then performed the nanotrap particles pulldown. Our western blot analysis demonstrated that reducing the samples allowed for the increased recovery of intracellular Nef by NT073 and NT080 and membrane-associated Nef by NT082, NT084, and NT086 (data not shown). Collectively, our results indicate that the nanotrap particles can be used to enrich both forms of HIV-1 Nef.

### Nanotrap particles capture whole HIV-1 virions

**Figure 3:**
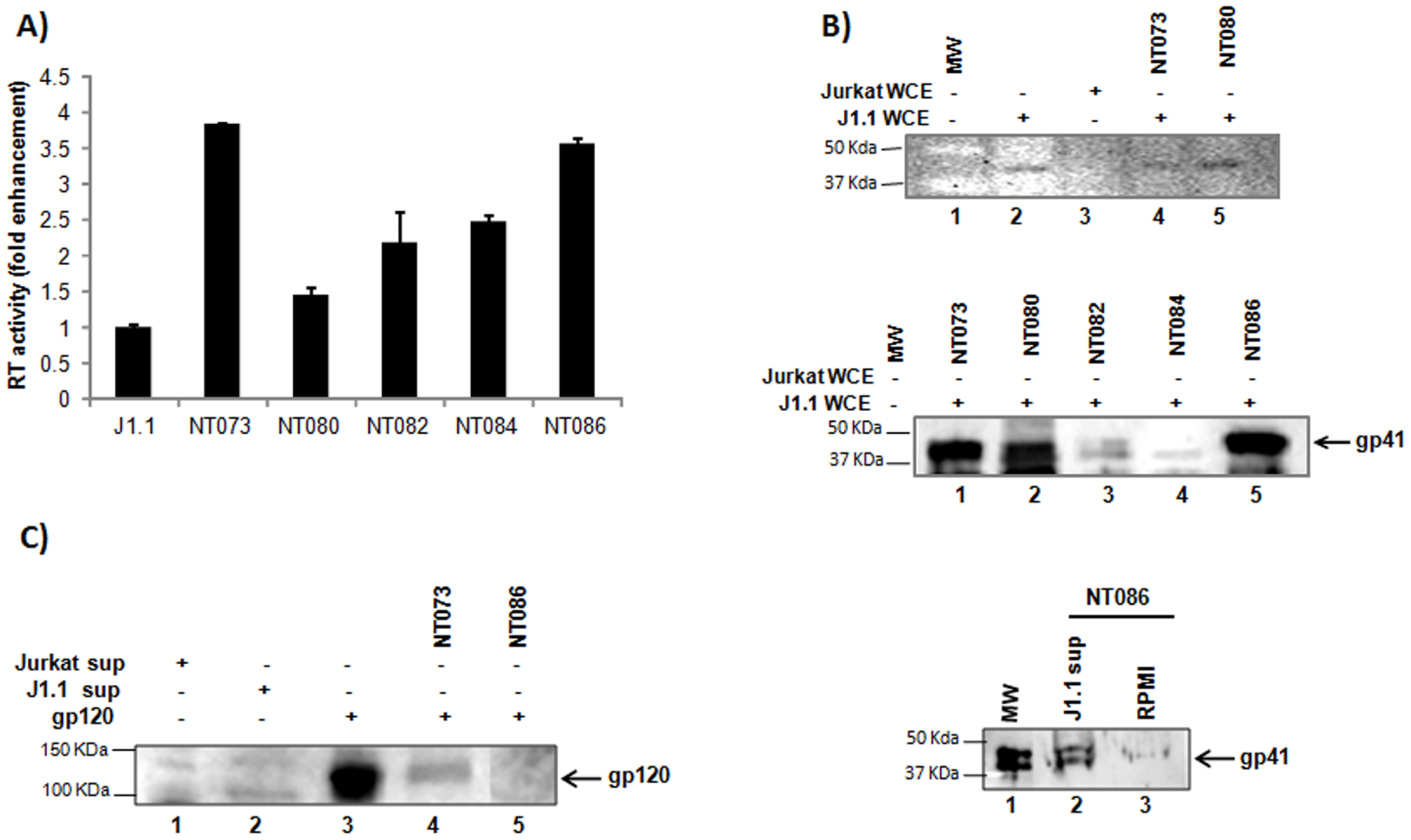
Capture of HIV-1 virions by nanotrap particles. (**A**) A 30% slurry of each nanotrap particle in unsupplemented RPMI (30 µl) was incubated with J1.1 cell supernatants (200 µl) for 30 min at room temperature. Virus-bound nanoparticles were washed to remove unbound virus, and the nanoparticle pellets were suspended in 50 µl of Tris (250 nM) + 1% Triton X-100, and assayed for RT activity without elution. Fold enrichment of virus binding to nanotrap particles was analyzed over J1.1 supernatant alone without prior enrichment. (**B**) HIV-1 gp41 was detected in J1.1 WCE by WB without nanoparticles pretreatment (upper panel, lane 2). The uninfected Jurkat WCE was used as negative control (lane 3). Binding of HIV-1 by nanotrap particles through interaction with gp41 was detected in J1.1 WCE following treatments with nanoparticles NT073 and NT080 (lanes 4 and 5, respectively). The captures of gp41 from J1.1 WCE were also shown by nanoparticles NT073, NT080 and NT086 but not by NT084 (middle panel). Fresh antibody was used for this panel. (**C**) Interaction of nanotrap particles with HIV-1 envelope gp120 was detected by western blot using anti-HIV human serum. Briefly, purified gp120 glycoprotein (1 µg) or J1.1 culture supernatant was spiked into water and incubated with or without nanotrap particles slurry for 30 min at room temperature, washed and suspended in Laemmli buffer before resolved on SDS-PAGE gel and analyzed by western blot.

Given the variable selectivity of nanotrap particles for binding multiple HIV-1 proteins, we next asked whether these particles could capture whole HIV-1 virions. Previously we have shown that NT053 particles efficiently captured Rift Valley Fever Virus (RVFV) and also protected the virions from proteolytic degradation [48]. To determine which nanotrap particle captures the highest level of HIV-1 virus particles, we incubated infected J1.1 cell supernatants with five different particles for 30 min at room temperature. After washing, the particles-bound HIV-1 virions were analyzed for RT activity. The results showed that both the NT073 and NT086 particles equally captured very high levels of HIV-1 virions, which enriched the RT activity by at least three times compared to the J1.1 supernatant without prior treatment of nanotrap particles. Both NT082 and NT084 exhibited a 2-fold increase in RT activity and NT080 showed the least activity. Interestingly, NT073 and NT082 nanotrap particles both have the same Cibacron Blue F3GA bait in the core, while the only difference is the presence of VSA within the particle shell. As shown in Figure 3A, NT073 which contains VSA bound almost double the HIV-1 particles compared to NT082, and hence implicate a role of the VSA shell in the capture of whole HIV-1 virions.

Because of its size (diameter ∼120 nm) HIV-1 virions may not completely enter into the core through the pores of the shell, we hypothesized that the nanotrap particles may bind the glycoproteins of the HIV-1 envelope, gp120 or gp41 without requiring the virions to enter into the particles. We utilized all five different nanotrap particles to investigate their capturing capacity of gp41. Standard slurry of each nanoparticle was added to J1.1 WCE, and assayed for the presence of gp41 by western blot (Fig. 3B upper panel). A band was observed with J1.1 WCE without nanoparticle treatment. Gp41 specific bands were also observed with NT073 and NT080 captured J1.1 WCE materials. In a repeated experiment, we also observed better gp41 binding with fresh antibody in the NT073, NT080 and NT086 captured J1.1 materials (Fig. 3B middle panel). Next, we asked whether NT086 could also interact with gp41 of cell-free HIV-1 in the J1.1 supernatant. As shown in Figure 3B (lower panel), the NT086 particles also bound to gp41 of the cell-free HIV-1 as evidenced by the right-size band on the western blot when stained with gp41 antibody. Taken together the above data indicate that nanotrap particles interacted with gp41 suggesting a mechanism of whole HIV-1 binding via gp41. We then spiked purified gp120 protein into FBS-supplemented RPMI and conducted the nanotrap particles pulldown. Following western blotting we observed that NT073 recovered approximately 25% of the input gp120 material, however the NT086 failed to capture any gp120 (Fig. 3C) even though it showed high levels of virus capture by RT assay (Fig. 3A). Collectively, the interactions between gp41 and the nanotrap particles especially NT073 and NT086 indicated a potential mechanism for capturing of intact HIV-1 particles by nanotrap particles.

### Captured virions maintain their infectivity

**Figure 4:**
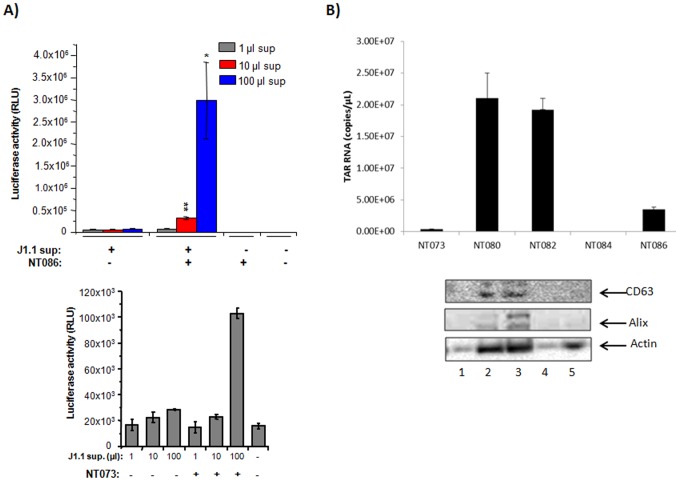
Capture of infectious HIV-1 virions by nanotrap particles. (**A**) HIV-1 infected J1.1 supernatants (1, 10, 100 µl) diluted to 1 ml in complete media, were either untreated or added to 50 µl of 30% slurry of NT086 nanotrap particles. The particles were incubated for one hour with gentle rotation at room temperature. The unbound virus was removed by centrifugation for 10 min before incubating the nanotrap particles with TZM-bl cells at 37*°C* in microtiter plates. Virus samples were also incubated with TZM-bl cells without prior treatment with nanotrap particles. After 48 hr post incubation, cells were lysed and HIV-1 transactivation analyzed by luciferase assay. The 2 asterices (p<0.01) and 1 asterix (p<0.05) represent the level of statistical significance between virus captures from 1 and 10 µl, and 10 and 100 µl supernatants, respectively. The NT073 nanoparticles were similarly used to capture infectious virions from J1.1 supernatants and analyzed by TZM-bl system as above (**B**) Exosomes (15 µl) collected from J1.1 cell supernatant were spiked into PBS (85 µl) and then incubated with a 30% slurry of five different nanotrap particles (30 µl) for 30 min. Nanotrap particles were washed and subjected to Trizol buffer for total RNA extraction. Evaluation of TAR-RNA contents of exosomes captured by nanotrap particles was performed via qRT-PCR using specific TAR-RNA primers. The five different nanotrap particles were similarly incubated with infected J1.1-derived exosomes and washed as in panel B. The nanoparticles-bound exosomes were then suspended in Laemmli buffer, separated on SDS-PAGE gel and analyzed by western blot using antibody to CD63 and Alix (standard markers for exosomes).

We asked whether the nanotrap particles could capture infectious HIV-1 particles. Since NT086 captured high levels of HIV-1 as measured by RT assay, and also bound to viral gp41, we utilized this particle to capture live HIV-1 from J1.1 culture supernatants. To assay for transcriptional activation of HIV-1, we utilized TZM-bl cells, a HeLa cell derivative with a stably integrated HIV-1 LTR-luciferase. These cells also express CD4 and both co-receptors CXCR4 and CCR5, which permits infection by both T- and M-tropic viruses [43]. We titrated these cells with equivalent volumes of the nanotrap pellets pre-treated with 1, 10 or 100 µl of J1.1 supernatant, and scored for transcriptional activation measured by luciferase assay. The unbound virus was removed from the particles by centrifugation before incubating with TZM-bl cells. Same amounts of J1.1 supernatants were also incubated with TZM-bl cells without prior treatment to compare the enrichment of HIV-1 binding by nanotrap particles. As shown in Figure 4A (upper panel), significantly higher levels of viral transactivation was observed with nanotrap particles-bound virus when compared with untreated virus (p<0.01 for 10 µl and p<0.05 for 100 µl) as measured by luciferase assay. Nanotrap particles alone did not show luciferase activity above background. Interestingly, in the same experiment, NT086 particles incubated with up to 20 µg of pcTat plasmid did not activate HIV-1 transactivation in TZM-bl cells (data not shown) indicating that DNA alone is neither capable of binding to nanoparticles and/or activating viral transcription. Using the same TZM-bl system, the capturing of infectious HIV-1 virions was also shown (Fig. 4A lower panel) by NT073, another nanotrap particle that bound highest levels of virions previously measured by RT assay (see Fig. 3A) indicating that certain nanotrap particles could consistently bind infectious HIV-1 virions. Collectively, these data indicate that nanotrap particles can significantly enrich for infectious HIV-1 particles potentially through an interaction with either the surface glycoprotein gp120 or gp41.

In order to validate the selectivity of NT073 and NT086 nanotrap particles for capturing HIV-1 virions, an additional control experiment was conducted where exosomes preparations from J1.1 supernatant were incubated with 5 different particles including the two above. Exosomes were first described by Harding et al. [49] as cup-shaped nanovesicles (30–100 nm) shed by a variety of cells. Exosomes usually incorporate certain host proteins including CD63, CD81, CD9, Alix, TSG101 and heat-shock proteins, and play important roles in intercellular communication, cellular inflammation, antigen presentation and cell death. Interestingly, the contents of exosomes can be altered by viral infections, and exosomes can also influence the outcome of the viral disease [50], [51], [52], [53]. We have recently observed that naïve cells exposed to HIV-1 exosomes containing miRNA are more susceptible to infection [46]. Since we have previously shown that exosomes from the HIV-1 infected J1.1 cells contain TAR-RNA [46], capturing of exosomes by nanotrap particles was quantified by analyzing the TAR-RNA copies in the captured exosomes by qRT-PCR and compared with the binding affinity for HIV-1 virions. Interestingly, both NT080 and NT082 particles captured exosomes containing up to 20 million copies of TAR-RNA while the highest HIV-1 capturing NT073 and NT086 particles bound exosomes at a minimum level (Fig. 4B upper panel). These data further indicate that the NT073 and NT086 nanotrap particles selectively captured HIV-1 particles.

To further validate the capture of exosomes from HIV-1 infected cell culture supernatant by nanotrap particles NT080 and NT082, the nanoparticles-bound exosomes materials were lysed in Laemmli sample buffer and separated on SDS-PAGE gels and then imunoblotted by antibodies to CD63 and Alix, standard marker proteins for exosomes. As shown in Figure 4B (lower panel), both CD63 and Alix were detected only in the NT080 and NT082 bound materials while the high level virions capturing nanoparticles NT073 and NT086 did not capture any exosomes indicating there is a strong selectivity for capturing virions or exosomes by nanoparticles.

### Capture of virions and exosomes from patients' sera

**Figure 5:**
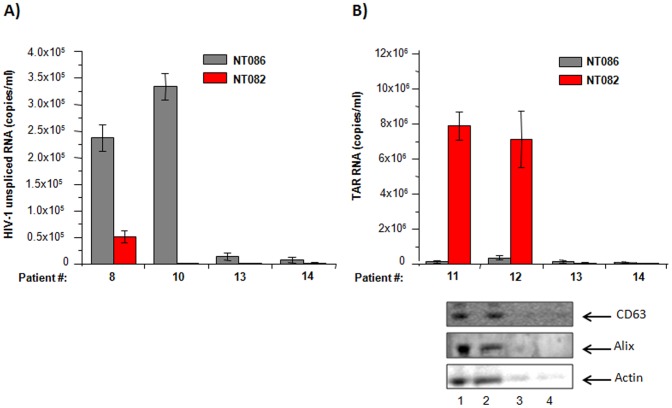
Capture of HIV-1 virions and exosomes containing TAR-RNA in patients' serum by nanotrap particles. Aliquots of serum samples from 4 HIV-1 infected HAART individuals (#8, #10, #11 and #12) and 2 uninfected donors (#13 and #14) were incubated separately with nanotrap particles (NT086 and NT082) for 30 min at room temperature. After removing unbound materials by washing, nanotrap particles were incubated with Trizol buffer for total RNA extraction. The levels of HIV-1 virions and exosomes containing TAR-RNA bound to each type of nanotrap particles were quantified by qRT-PCR using primers specific to HIV-1 unspliced RNA (**A**) and TAR-RNA (**B**). The similarly prepared nanotrap particles-bound serum-derived materials (from samples #11, #12, #13 and #14) were also suspended in Laemmli buffer, separated on SDS-PAGE gel, and analyzed by western blot using antibody to CD63 and Alix (standard markers for exosomes) (see lower panel).

We were interested to see whether the nanotrap particles NT086 that was found to capture HIV-1 virions from cell culture supernatant at high levels could also selectively capture HIV-1 directly from infected human serum samples. Also, the NT082 particles that captured high levels of exosomes (containing TAR-RNA) prepared from HIV-1 infected culture supernatant was used as control to verify the selective affinity of nanotrap particles for virions or exosomes. As shown in Figure 5A, NT086 selectively captured HIV-1 virions from two different HAART patients' sera (#8 and #10) at a level thousand-fold higher than the NT082 that was shown to selectively capture exosomes at much higher level than NT086. As expected when nanoparticles were used to capture exosomes-containing TAR-RNA from the HIV-1 infected serum samples (#11 and #12), once again the NT082 selectively captured exosome particles at levels more than a million-fold higher than the highest-level HIV-1 capturing NT086 (Fig. 5B upper panel). These data further indicate that some nanotrap particles can be used to selectively capture both HIV-1 virions and exosomes containing TAR-RNA directly from patients' sera.

In order to further validate the capture of exosomes from HIV-1 infected serum samples (#11 and #12), the nanoparticles-bound materials were lysed by Laemmli sample buffer, separated on SDS-PAGE gel and immunoblotted with antibody to exosome marker proteins CD63 and Alix. Interestingly again, CD63 and Alix were present only in the serum samples where the TAR-RNA was detectable by NT082 nanoparticles (Fig. 5B lower panel) indicating further the selectivity of nanoparticles for virions or exosomes.

### Nanoparticles capacity to bind whole virions and exosomes

**Figure 6:**
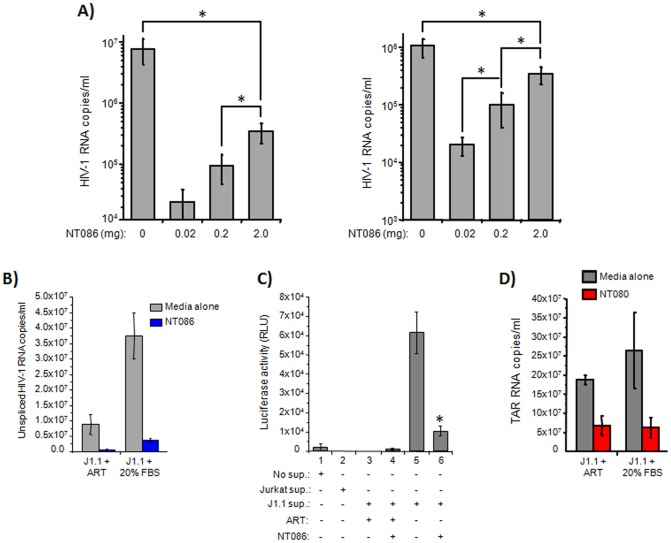
HIV-1 and exosomes capturing capacity of nanoparticles. (**A**) 100 µl aliquot of the dual-tropic HIV-1 89.6 containing 10 or 1 ng p24/ml was incubated with a pellet of 0.02, 0.2 or 2.0 mg of NT086 for 30 min at room temperature. The virus bound-nanoparticles were washed and RNA isolated. The RNA was converted to cDNA and PCR reaction mixtures were prepared using high-low concentrations of cDNA, Gag primers and the iTaq Universal SYBR Green Supermix. Serial dilutions of DNA from 8E5 cells were used as the standards. Quantitative real-time PCR reactions were carried out in triplicate. The asterix represents the statistical significance at the level of p<0.05. (**B**) The infected J1.1 cells were treated with or without anti-retrovirals (ART), and then sups were incubated with NT086 to capture virions. After washing, the total RNA was extracted from the nanoparticle captured materials and then subjected to qRT-PCR using specific primers to unspliced HIV-1 RNA. (**C**) The infectivity of the nanoparticles captured virions were also analyzed by incubating them with TZM-bl cells and measuring luciferase activity. The asterix represents the statistical significance at the level of p<0.05. (**D**) The NT080 nanoparticles capture of exosomal RNA from J1.1 supernatants with or without pretreatment of ART were similarly measured by qRT-PCR using specific primers to TAR RNA.

Following establishing the selectivity of certain nanoparticles for virion capture, we tested the concentration threshold of nanoparticles for maximum capture of HIV-1 virions. Using three different concentrations of NT086 (0.02, 0.2 and 2.0 mg) and two concentrations of HIV-1 89.6 virions (1×10^6^ and 10×10^6^ RNA copies) in a standard virus capture protocol, it was observed that the virus binding was directly correlated to the nanoparticles concentration, and the 2.0 mg nanoparticles (the highest concentration used) captured about 30% of the total input virus (the highest levels of virus capture) (Fig. 6A). Interestingly, there was not much difference in virus binding between the two different virus inputs. Nevertheless, these results suggest that the concentration lower than the 2.0 mg of nanoparticles can be used to efficiently capture a high percentage of virions from a more diluted HIV-1 infected samples. In order to mimic the nanoparticles capture of virions and exosomes from a latently infected sample, we treated the infected J1.1 cells with an anti-retroviral cocktail (ART) containing two RT inhibitors and one protease inhibitor for 10 days, before using the supernatants for capture by nanoparticles. The rationale here is that the J1.1 cells normally secret virus in the supernatant, and in the presence of ART, the level of extracellular virus is decreased. On the other hand, addition of 20% FBS after removal of ART, will increase the extracellular virus levels. First, we tested the capture of ART-treated supernatants by NT086 nanoparticles for both HIV-1 RNA copies by qRT-PCR and infectious virions by TZM-bl system. As shown in Figure 6B, the level of viral RNA copies in the J1.1 supernatant were markedly reduced by ART treatment compared to untreated supernatant, and as expected the NT086 capture higher amount of HIV-1 virions from untreated samples. Similarly, the ART treatment entirely eliminated the infectious virions (Fig. 6C, lanes 3), and thus there was no capture of infectious virus by NT086 nanoparticle (lane 4). However, the highest levels of infectious virus was recovered from the supernatant in the absence of ART (lane 5) while approximately, 20–25% of infectious virus in the J1.1 supernatant was captured by NT086 nanoparticles (lane 6). We also determined the level of exosomes capture in the culture supernatants by NT080 following treatment of infected J1.1 cells with or without ART. As shown in the Figure 6D, NT080 captured approximately 30% of the total input of exosomes when compared with no nanoparticles pretreatment suggesting once again the nanoparticles could more efficiently capture exosomal RNA in a more diluted sample.

## Discussion

Given the staggering global estimates for newly acquired HIV-1 infections each year, early detection of infection is of paramount importance for both successful treatment and prevention of the disease. In an effort to diagnose HIV-1 infection earlier, novel detection techniques are continuously being developed and improved upon with the aim of increasing the sensitivity of the assay as well as reducing the complexity, time and cost. It is also evident that a growing demand for new technologies has led to the development of several HIV-1 detection methods with great promise, particularly for resource limited settings. However, none of these methods have shown to capture, sequester and concentrate HIV-1 virions or viral proteins. Furthermore, the flexibility of new technology to combine with existing assays is often limited.

In the current manuscript, we employed a novel detection technology using hydrogel nanotrap particles [54] as a unique means to concentrate target molecules related to HIV-1 infection. The advantages of using nanotrap particles include versatility and selectivity in binding of various target molecules as well as compatibility to multiple buffering conditions used in standard assays including western blot, RT, ELISA, PCR, etc. In addition, nanotrap particles reactions require a small sample volume (e.g., <1 ml) and a short incubation at room temperature (e.g., 30 min) even though the volume requirement can easily be scaled up if it is necessary to concentrate target molecules from a large volume of diluted material. This is consistent to the previous report that demonstrated successful capture of biomarkers in urine or blood within 30 min [54]. More importantly, nanoparticle chemistries can be designed to selectively capture and concentrate small molecules with low abundance in complex body fluids that are usually undetectable by the standard assays [37]. Furthermore, nanotrap particles-captured molecules could be protected from degradation by proteolysis [37], [48]. Thus, the nanotrap particles technology potentially offers multiple advantages such as rapidity, simplicity and high sensitivity over the existing assays of HIV-1 detection.

Our objective was to determine whether the nanotrap particles could successfully capture HIV-1 virions or proteins in cell culture media with a long-term goal of capturing specific target molecules in complex body fluids including human serum, cerebrospinal fluid, urine, etc. Here we demonstrate that several of the nanotrap particles (see Table 1) effectively captured HIV-1 proteins, including the viral transactivator Tat, Nef and gp41. Moreover, we showed that the capture of each target molecule by nanotrap particles was selective and can be detectable by standard assay platforms including western blotting, reverse transcriptase and PCR. Interestingly, we have demonstrated that the bait Cibacron Blue in NT082 successfully captured Tat_36-72_ peptide but was unable to capture p24 or Nef implicating selectivity of nanotrap particles for a specific target molecule. Furthermore, the nanotrap particles NT086 containing the bait Methacrylate bound high amount of whole HIV-1 particles as well as glycoprotein gp41 and membrane-associated Nef protein while completely failed to capture intracellular Nef and p24. Although the precise interaction between NT086 and HIV-1 virions is yet to be determined, these data suggest that the bait Methacrylate may have strong selectivity for HIV-1 glycoprotein or proteins associated with cell membrane. The selective binding of HIV-1 glycoprotein by the NT086 particles was further supported by the fact that exosomes derived from the same HIV-1 infected cells were only minimally captured by the NT086 while they were selectively captured by the NT080 and NT082 particles at high levels. These data further suggest that even though both HIV-1 and exosomes were derived from the infected J1.1 cells and likely contained host membrane proteins, the nanotrap captures were very selective for each target molecule. More importantly, the nanotrap particles NT086 and NT082 selectively captured HIV-1 virions and exosomes containing TAR-RNA, respectively, from HAART patients' serum samples.

The most important finding of this study was that the nanotrap particles could capture cell-free infectious HIV-1 virions from infected culture supernatant and that can be efficiently detected in susceptible TZM-bl reporter cells. In HAART patients similar to long-term non-progressors (LTNP) or elite suppressors (ES), plasma virus levels are usually very low (e.g., <50–1000 copies/ml of blood) but can be detectable by RT-PCR [55], [56], [57], [58]. However, recovery of infectious virus from these patients in cell culture could be difficult, expensive and time consuming [19], [59]. Our current data suggest that the nanotrap technology could provide a valuable and cost effective alternative to determine the infectious status of blood samples from HAART patients, LTNP or ES.

**Figure 7:**
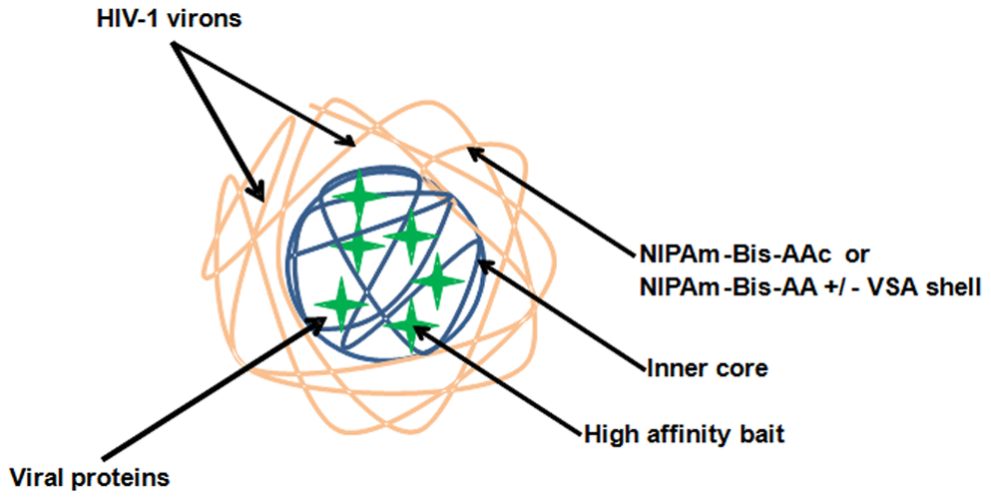
Schematic diagram of a nanotrap particle. The outer porous shell, inner core and the affinity baits in the core are represented by yellow, blue and green, respectively. In some nanotrap particles vinyl sulfonic acid (VSA) is incorporated into the outer shell. The smaller viral proteins readily enter into the particle core through the pores of the shell and bind to the specific affinity baits. The larger viral particles do not completely enter into the core of the nanoparticle, and thus bind to the outer shell of the particles.

The nanotrap particles employed in this study were approximately 700–800 nm in diameter, and composed of a porous shell matrix (approximately 170 nm thick) covalently functionalized with high affinity bait of approximately 300 nm in diameter core [37]. The sieving shell of each nanotrap particle serves to restrict entry of high abundance, high molecular weight carrier proteins like albumin in serum while allowing entry of low abundance smaller molecules. The diverse high affinity bait contains bulky aromatic rings with a variety of functional groups. Thus the baits function in varying capacities to bind and dissociate the target molecules from carrier proteins within the sample through electrostatic and hydrophobic interactions [38], [60]. As shown in a general scheme of nanotrap particles (Fig. 7), although the mechanism of HIV-1 interaction is not yet clear, smaller viral proteins (Tat, Nef or gp41) may likely enter through the shell pores and bind to specific bait while the larger HIV-1 virions (100–120 nm) may not require entering into the particles to be captured. Once captured, the smaller target molecules are sequestered within the core, and likely be protected from degradation [48].

Finally, we have identified specific nanotrap particles relevant to the detection of infectious HIV-1 virions or proteins such as Tat, Nef or gp41. Also, we have established the working conditions compatible with downstream assays including TZM-bl cell system, western blot, RT and qRT-PCR. Currently, a Viral Outgrowth Assay (VOA) [12] is used to quantify latent reservoirs in PBMCs from HIV-1 infected HAART patients. This assay involves purification of resting CD4+T cells from patients' PBMCs and then co-cultivation of the purified primary T-cells with the MOLT-4/CCR5 cell line to allow replication of the latent provirus that is measured by qRT-PCR specific for HIV-1 RNA. Our current assay will provide a valuable alternative to the VOA where nanotrap particles will capture majority of the HIV-1 particles from patient's serum and allow the detection of the replicating virus in the reporter TZM-bl cells. Collectively, our results lend support to the use of nanotrap particles as a reliable means for capturing and detecting HIV-1 virions and viral proteins from complex biological fluids *in vivo*.
